# Anti-inflammatory effects of *Viola yedoensis* and the application of cell extraction methods for investigating bioactive constituents in macrophages

**DOI:** 10.1186/s12906-016-1142-9

**Published:** 2016-06-14

**Authors:** Yun Hee Jeong, You-Chang Oh, Won-Kyung Cho, Hyeji Shin, Ki Yong Lee, Jin Yeul Ma

**Affiliations:** Korean Medicine (KM)-Application Center, Korea Institute of Oriental Medicine, 70, Cheomdanro, Dong-gu, Daegu, 41062 Republic of Korea; College of Pharmacy, Korea University, 2511, Sejongro, Sejong, 30019 Republic of Korea

**Keywords:** *Viola yedoensis* ethanol extract, Nuclear factor-kappaB, Mitogen-activated protein kinase, Heme oxygenase-1, Cell extraction, Bio-active components

## Abstract

**Background:**

*Viola yedoensis* (VY, Violaceae) is a popular medicinal herb used in traditional eastern medicine for treating lots of diseases, including inflammation and its related symptoms. However, the anti-inflammatory properties of VY have not been demonstrated. In the present study, we investigated the anti-inflammatory effects of VY ethanol extract (VYE) on macrophages and attempted to identify the bioactive components of VYE.

**Methods:**

We assessed the effects of VYE on secretion of nitric oxide (NO) and inflammatory cytokines such as tumor necrosis factor (TNF)-α, interleukin (IL)-6, and IL-1β. In addition, we explored the expression of inducible nitric oxide synthase (iNOS), cyclooxygenase (COX)-2, and changes in heme oxygenase (HO)-1, nuclear factor (NF)-kB, and mitogen-activated protein kinase (MAPK) signaling pathways in RAW 264.7 macrophages stimulated by lipopolysaccharide (LPS). In addition, a rapid and useful approach to identify potential bioactive components in VYE with anti-inflammatory effects was developed using murine macrophage cell extraction coupled with high-performance liquid chromatography tandem mass spectrometry (LC-MS).

**Results:**

We found that VYE exerted anti-inflammatory activity by inhibiting the production of key inflammation mediators and related products, as well as suppression of HO-1, NF-kB, and MAPK signaling pathway activation in RAW 264.7 cells. In addition, we identified two compounds in VYE via the cell extraction method.

**Conclusions:**

Our results revealed that VYE exerts anti-inflammatory activities and its detailed inhibitory mechanism in macrophages. Furthermore, we identified bioactive components of VYE.

## Background

VY is one of the traditional medicinal herb that belongs to the violet family of Violaceae, called “Hojebigot” in Korea. The dried aerial component of VY is Viola Herba, and is produced in the southern regions of Korea, China, and Japan. VY is a well-known herb in traditional oriental medicine used for the treatment of inflammation-related diseases including swelling, sores, boils, furuncles, carbuncles, snakebites, and acute and chronic hepatitis [1]. Recent studies have shown that VY has biological activities and pharmacological functions such as anti-HIV, anti-coagulant activities, and protective effects against LPS-induced acute lung injury in mice [2–4]. However, the detailed molecular mechanism of the anti-inflammatory effects of VY is not well-characterized.

Inflammation is a first host immune response to protect the body from injury or infection. Normal inflammatory reactions are self-limited by down regulating pro-inflammatory proteins and increasing anti-inflammatory mediators [5, 6]. The onset of chronic diseases—such as inflammatory arthritis, vascular diseases, and cancer—is closely associated with uncontrolled inflammatory responses or overproduction of inflammatory mediators [7].

Macrophages play a crucial role during inflammation by regulating immune responses. Macrophages activated by various stimulants can generate a broad array of pro-inflammatory mediators, such as NO, iNOS, COX-2, TNF-α, IL-6, and IL-1β [8–10]. Inflammatory cytokine production and release in response to LPS is mediated by the activation of NF-kB and MAPK in macrophages [11, 12]. NF-kB and MAPKs are typical inflammatory signaling pathways in macrophages. These two pathways induce pro-inflammatory cytokines and release a wide range of inflammatory mediators. Therefore, the majority of targets for the development of therapeutic approaches to treat various inflammatory diseases are associated with inhibition of these pathways.

In an unstimulated state, p65 of NF-kB is sequestered by inhibitors of NF-kB alpha (IkBα) in the cytoplasm. Activation of NF-kB by inflammatory stimulants, such as LPS, occurs via phosphorylation and degradation of IkBα. Phosphorylated IkBα is dissociated from the p65/IkBα complex and free NF-kB translocates into the nucleus [13], where it regulates several genes important for immunity, including iNOS, COX-2, and certain cytokines [14–16]. MAPK consists of extracellular signal-regulated kinase (ERK), Jun NH_2_-terminal kinase (JNK), and p38. MAPKs play a critical role in delivering inflammatory signals from the extracellular region to the intracellular region or nucleus [17]. MAPK is activated by phosphorylation of its component pathways to thereafter activate the NF-kB pathway and iNOS gene expression.

NO is synthesized from L-arginine by iNOS, whose expression is closely associated with the induction of HO-1. HO-1 is also one of the important regulator of inflammation and has exhibited an essential role in protecting the body from inflammatory processes [18]. Upon activated macrophages, HO-1 and carbon monoxide (CO) have been revealed to exert anti-inflammatory effects through decrease the expression of pro-inflammatory mediators including NO, PGE_2_ and cytokines [19, 20]. Thus, enhanced the production of HO-1 expression may result in increased a lot of therapeutic agents.

To screening a bioactive components in a conventional procedures, it have been performed through a complex process, including isolating and purification of ingredient from natural products. Modern pharmacological researches have been investigated the potential biological activity of natural resources using the several new techniques, such as cell membrane chromatography, biomembrane affinity chromatography and cell extraction coupled with analytical techniques. We proposed a method of cell extraction coupled with LC-MS analysis to screen for bioactive components using macrophages. These techniques involve direct treatment of the natural products in the cells, incubation for 24 h to allow the bioactive components to selectively combine with specific receptors and/or channels and/or enzymes on the cell membrane or inside cells, extraction with 70 % acetonitrile, and detection of the cell-combining components by LC-MS analysis. Recently, these methods were employed for screening of single compounds or drug candidates from natural resources used in traditional medicine [21, 22].

In present study, we examined the anti-inflammatory effects of VYE on LPS-induced inflammation in RAW 264.7 and mouse primary macrophage cells. In addition, we evaluated whether VYE regulates NF-kB, MAPK, and HO-1 pathways to demonstrate its inhibitory mechanism underlying its anti-inflammatory effect. Furthermore, we explored the bioactive components of VYE using cell extraction.

## Methods

### Preparation of VYE

VY was obtained as a dried herb from Yeongcheonhyundai Herbal Market (Yeongcheon, Korea) and verificated by Prof. KiHwan Bae (Chungnam National University, Daejeon, Korea). The voucher specimen of VY was stored in the herbarium of KM-Application Center, Korea Institute of Oriental Medicine. The pulverized plant (30.0 g) was extracted with 390 mL of 70 % ethanol at 40 °C in a shaking incubator (100 rpm) for 24 h. After extraction, the solution was filtered using 185-mm filter paper (Whatman, Piscataway, NJ, USA) and enrichmented using a rotary vacuum evaporator (Buchi, Tokyo, Japan). Samples were then freeze-dried and maintained in desiccators at −20 °C before use. The yield of VYE was 11.25 %.

### Materials and reagents

Roswell Park Memorial Institute (RPMI) 1640 medium, antibiotics, and fetal bovine serum (FBS) were obtained from Hyclone (Logan, UT, USA). All cell culture dishes and plates were obtained from SPL life sciences (Pocheon, Korea). LPS, dexamethasone, bovine serum albumin (BSA), cell counting kit (CCK), and enzyme-linked immunosorbent assay (ELISA) antibody sets were obtained from Sigma (St. Louis, MO, USA), Dojindo (Kumamoto, Japan), and eBioscience (San diego, CA, USA), respectively. Various primary antibodies for Western blot analysis were purchased from Cell Signaling Technology (Boston, MA, USA). Horseradish peroxidase (HRP)-conjugated secondary antibodies were obtained from Thermo Scientific (Rockford, IL, USA). An extraction kit for RNA isolation was purchased from iNtRON (Sungnam, Korea). DNA synthesis kits, oligonucleotide primers, and AccuPower® 2X Greenstar qPCR Master Mix (ROX) were obtained from Bioneer (Daejeon, Korea).

### Macrophage cell culture and treatment of test drug

The murine macrophage RAW 264.7 was obtained from the American Type Culture Collection (ATCC, Manassas, VA, USA) and maintained in RPMI 1640 (contained 10 % FBS and antibiotics). Cells were then incubated in humidified 5 % CO_2_ atmosphere at 37 °C. To stimulate the inflammatory signals of macrophages, the RPMI 1640 medium was exchanged for fresh medium and 200 ng/mL of LPS was added in the presence or absence of VYE (10, 30, 50, 100 and 150 μg/mL) for the indicated periods.

### Isolation of mouse peritoneal macrophage and cell culture

Male BALB/c mice (25 ± 3 g) were obtained from SamtakoBioKorea (Osan, Korea). 300 μL of sterilized 3 % sodium thioglycollate (Sigma, St. Louis, MO, USA) was injected to abdominal cavity of mice. All mice were housed at 5 per cage at room temperature (RT) under a 12-h: 12-h light/dark cycle. After three days, the mice were killed and peritoneal macrophages were collected by washing peritoneal cavity with 10 mL (per each mouse) of ice-cold phosphate-buffered saline (PBS). The cells were centrifuged at 2000 rpm for 5 min at 4 °C and the supernatant containing red blood cell (RBC) debris was discarded. The cell pellet was resuspended in completed RPMI 1640 and incubated for 18 h for attachment to the culture plate [23]. To stimulate the cells, the medium was exchanged for fresh RPMI 1640 and LPS was added in the presence or absence of VYE for 24 h. Animal study was performed according to the Guide for the Animal Care and Use Committee of the Korea Institute of Oriental Medicine (reference numbers: 14–079).

### Cell viability assays

RAW 264.7 macrophages were mechanically scraped and plated into 96 well culture plates (5 × 10^4^ cells/well). After 18 h, five concentrations of VYE were added to the cells and incubated for 24 h. CCK solutions (10 μL) were applied to each well. Then the cells were incubated for a further 1 h. The optical density value was readed at 450 nm using an ELISA reader (infinite M200, Tecan, Männedorf, Switzerland).

### Measurement of NO production

To measure secretion of NO, cells were plated into 96 well culture plates (5 × 10^4^ cells/well). After 18 h, cells were treated with VYE and stimulated using LPS for 24 h. Griess reagent (1 % sulfanilamide, 0.1 % N-1-napthylethylenediamine dihydrochloride, and 2.5 % phosphoric acid) was added to each well and further incubated at RT for 5 min [24]. Absorbance at 570 nm was determined using the ELISA reader.

### Determination of inflammatory cytokines

To explore the inhibitory effects of VYE on cytokine production in macrophages upon LPS stimulation, the secretion of TNF-α, IL-6, and IL-1β was measured using an ELISA antibody set. Macrophages were grown in 24 well culture plates (5 × 10^4^ cells/well). The cells were pretreated with VYE for 1 h and then challenged with LPS for another 24 h. The supernatants were then centrifuged at 13000 rpm at 4 °C for 5 min for discarded cell debris. The levels of cytokine were analyzed using ELISA antibody sets, according to protocol of manufacturer.

### Preparation of whole cell, cytoplasmic and nuclear fractions

To measure protein expression, cells were stimulated by LPS treatment in the presence or absence of VYE for the indicated periods (30 min–24 h). After incubation with VYE and/or LPS, cells were collected and resuspended in radio immunoprecipitation assay (RIPA) buffer (Millipore, Bedford, MA, USA) containing inhibitor cocktail (protease and phosphatase inhibitor) to get whole cell lysates. Cytoplasmic and nuclear lysates were isolated by NE-PER nuclear and cytoplasmic extraction reagents (Thermo Scientific, Rockford, IL, USA) according to protocol of manufacturer.

### Western blot analysis

Total proteins of whole cell lysates or cytoplasmic and nuclear extracts were assayed using Bradford’s reagent (Bio-Rad Hercules, CA, USA). The proteins were divided using sodium dodecyl sulfate-polyacrylamide gel electrophoresis (SDS-PAGE) and transferred to a nitrocellulose (NC) membrane (Millipore, Bedford, MA, USA). After blocking of nonspecific sites with 3 % BSA, the membranes were incubated with each primary antibodies at RT 2 h or 4 °C overnight. The membranes were subsequently incubated with HRP-conjugated anti-mouse or anti-rabbit secondary antibodies. The signals were detected using Super Signal West Femto chemiluminescent substrate (Thermo Scientific, Rockford, IL, USA).

### Total RNA extraction and real-time reverse transcription-polymerase chain reaction (real-time RT-PCR)

Total cellular RNA was extracted by easy-BLUE™ RNA extraction kit (iNtRON Biotech, Daejeon, Korea) in accordance with the protocol of manufacturer. cDNA was synthesized from 1 μg of total RNA using AccuPower®CycleScript RT PreMix (Bioneer, Daejeon, Korea). The oligonucleotide primers for real-time RT-PCR are indicated in Table 1 [25]. The samples were setup in triplicate in a 20 μL total volume: 0.3 μM final concentrations of each primer (1 μL of forward and reverse primer), 10 μL of AccuPower® 2X Greenstar qPCR Master Mix (Bioneer, Daejeon, Korea), 3 μL of 0.1 % DEPC-treated water, and 5 μL of template DNA. The following PCR conditions were applied: TNF-α, IL-6, IL-1β, iNOS, COX-2, HO-1, and β-actin, with 40 cycles of 94 °C for 15 s and 60 °C for 1 min [25]. The amplification and analysis were performed by a QuantStudio 6 Flex Real-time PCR System (Thermo Scientific, Rockford, IL, USA). Samples were compared by the relative CT method. The results of real-time RT-PCR were presented as inflammatory mediator gene induction fold, which were calculated using β-actin as an internal control.

**Table 1:** Primers used for real-time RT-PCR analysis

Target gene	Primer sequence
TNF-α	F: 5’-TTCTGTCTACTGAACTTCGGGGTGATCGGTCC-3’
	R: 5’-GTATGAGATAGCAAATCGGCTGACGGTGTGGG-3’
IL-6	F: 5’-TCCAGTTGCCTTCTTGGGAC-3’
	R: 5’-GTGTAATTAAGCCTCCGACTTG-3’
IL-1β	F: 5’-ATGGCAACTGTTCCTGAACTCAACT-3’
	R: 5’-CAGGACAGGTATAGATTCTTTCCTTT-3’
iNOS	F: 5’-GGCAGCCTGTGAGACCTTTG-3’
	R: 5’-GCATTGGAAGTGAAGCGTTTC-3’
COX-2	F: 5’-TGAGTACCGCAAACGCTTCTC-3’
	R: 5’-TGGACGAGGTTTTTCCACCAG-3’
HO-1	F: 5’-TGAAGGAGGCCACCAAGGAGG-3’
	R: 5’-AGAGGTCACCCAGGTAGCGGG-3’
β-actin	F: 5’-AGAGGGAAATCGTGCGTGAC-3’
	R: 5’-CAATAGTGATGACCTGGCCGT-3’

*F* forward, *R* reverse

### Cell extraction

RAW 264.7 cells were seeded into 100-mm culture dishes at a density of 1.0 × 10^6^ cells/mL, grown in completed RPMI medium, and maintained at 37 °C in an atmosphere of 5 % CO_2_ for 18 h. The culture medium was discarded, and serum-free RPMI medium and VYE (at a final concentration of 150 μg/mL) were added, followed by incubation for 24 h. The cells were then harvested and centrifuged at 2000 rpm for 5 min, after which the deposited cells were washed five times with PBS to remove uncombined components. Finally, the deposited cells were extracted with 1 mL of 70 % acetonitrile by ultrasonic extraction for 1 h. After centrifugation at 13,000 rpm for 10 min, the obtained supernatant was filtered through 0.45 μm nylon membranes for LC-MS analysis.

### The application of HPLC coupled with Q-TOF mass spectrometry

Agilent 1260 series (Agilent, Santa Clara, CA, USA) was used for chromatography analysis with an online degasser, a binary pump, an auto plate-sampler, a thermostatically controlled column compartment, and a UV detector. Chromatographic separation was achieved on an Shiseido CapCell PAK C18 column (150 × 4.6 mm, 5 μm) by gradient elution of a mixture of water (solvent A) and acetonitrile (solvent B) containing 0.1 % formic acid at a flow rate 0.6 mL/min. The gradient elution system was as below: 0–5 min, 15 % B; 5–30 min, 15–95 % B. A tandem quadrupole time-of-flight (Q-TOF) mass spectrometery was performed on an Agilent 6530 Q-TOF mass spectrometer (Agilent, Santa Clara, CA, USA). The acquisition parameters were set as follows. The analysis was operated in negative ion mode with an electrospray ionization (ESI) interface. The nebulizer pressure was set to 40 psi. The voltages of capillary, fragmentor, and skimmer were set to 4000 V, 175 V, and 65 V, respectively. The gas used for both drying and sheath was nitrogen. The temperature and flow rate of gas for each process were 325 °C; 10.0 L/min and 350 °C; 12.0 L/min. The mass scan range was configured from 50 to 1000 *m/z* and all data were collected in centroid mode. The accurate-mass capability of the TOF analyzer allowed reliable confirmation of the identity of the detected metabolites, normally with mass errors below 5 ppm in routine analysis. The MassHunter Workstation software LC/MS Data Acquisition for 6530 series Q-TOF (version B.05.00) was used to adjust the parameters for acquiring data.

### Extraction, fractionation and isolation

Dried whole plants of VY (600.0 g) were ground and extracted three times with 70 % ethanol for 90 min using an ultrasonicator. The solvent was then evaporated under vacuum. The residue (58.2 g) was suspended in water and then partitioned with *n*-hexane, EtOAc, and *n*-BuOH, successively. The *n*-BuOH fraction (16.5 g) was separated into six fractions (VY 2A-2 F) on an HP-20 column chromatography using gradient elution with H_2_O 100 % to MeOH 100 %. According to the TLC profiles, fraction VY-2D was re-chromatographed on RP C_18_ column chromatography using gradient elution with H_2_O-MeOH (2:1 to 3:2) to give Compound **1** (273 mg) and Compound **2** (226 mg). A summary of the extraction, fractionation, and isolation scheme is shown in Fig. 9.

### Compound 1; Isoschaftoside

ESI-MS *m/z* 563.1441 [M-H]^−^. ^1^H-NMR (600 MHz, DMSO-d_6_): δ 8.03 (2H, d, J = 8.5 Hz, H-2′ and H-6′), 6.92 (2H, d, J = 8.5 Hz, H-3′ and H-5′), 6.82 (1H, s, H-3), 4.76 (1H, d, J = 9.5 Hz, Glc-H-1), 4.72 (1H, d, J = 9.8 Hz, Ara-H-1), 3.25–3.90 (9H, m, Ara-H-2-5 and Glc-H-2-6) ppm. ^13^C-NMR (150 MHz, DMSO-d_6_):δ 61.3 (Glc-C-6), 68.5 (Ara-C-4), 69.6 (Ara-C-2), 70.2 (Ara-C-5), 70.6 (Glc-C-4), 71.0 (Gla-C-2), 73.3 (Glc-C-1), 73.9 (Ara-C-3), 74.2 (Ara-C-1), 78.9 (Glc-C-3), 81.9 (Glc-C-5), 102.6 (C-3), 103.7 (C-10), 105.8 (C-8), 108.1 (C-6), 116.0 (C-3′ and C-5′), 121.2 (C-1′), 129.1 (C-2′ and C-6′), 155.1 (C-9), 158.3 (C-5), 161.1 (C-7), 161.3 (C-4′), 164.1 (C-2), 182.4 (C-4) ppm.

### Compound 2; Apigenin 6, 8-di-C-α-L-arabinopyranoside

ESI-MS*m/z* 533.1363 [M-H]^−^. ^1^H-NMR (600 MHz, DMSO-d_6_): δ 8.02 (2H, d, J = 8.4 Hz, H-2′ and H-6′), 6.91 (2H, d, J = 8.4 Hz, H-3′ and H-5′), 6.83 (1H, s, H-3), 4.70 (1H, d, J = 9.5 Hz, Ara-H-1′), 4.70 (1H, d, J = 9.5 Hz, Ara-H-1″), 3.44–3.89 (10H, m, Ara-H-2′-5′ and Ara-H-2″-5″) ppm. ^13^C-NMR (150 MHz, DMSO-d_6_):δ 182.3 (C-4), 164.2 (C-2), 161.5 (C-7), 161.2 (C-5), 161.2 (C-4′), 155.0 (C-9), 129.5 (C-2′ and C-6′), 121.1 (C-1′), 116.0 (C-3′ and C-5′), 108.3 (C-6), 104.9 (C-8), 103.4 (C-10), 102.3 (C-3), 74.1 (Ara-C-1′), 74.1 (Ara-C-1″), 74.0 (Ara-C-3′), 74.0 (Ara-C-3″), 70.2 (Ara-C-5′), 70.2 (Ara-C-5″), 69.0 (Ara-C-2′), 69.0 (Ara-C-2″), 68.6 (Ara-C-4′), 68.6 (Ara-C-4″) ppm.

### Statistical analysis test

All data are presented as means ± SD of three independent experiments unless stated otherwise. Significant differences were determined using Student’s t-tests after comparing each treated group to the LPS group. Each experiment was repeated three times or more to yield comparable results. Values of * *P* < 0.01 and ** *P* < 0.001 were considered to indicate statistical significance.

### Statistical analysis software

GraphPad Prism version 5.02 software (GraphPad Software, Inc., San Diego, CA) was used for all the statistical analyses in this study.

## Results

### Effect of VYE on cell viability and NO production in RAW 264.7 cells

**Fig. 1:**
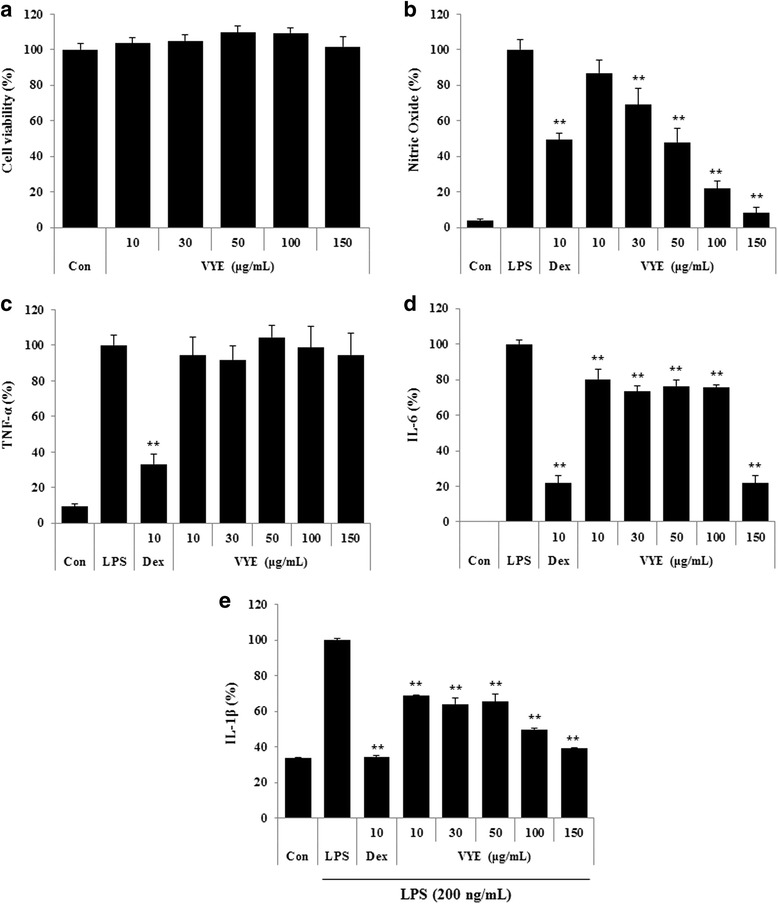
**a** Cytotoxicity of VYE in RAW 264.7 macrophages and the suppressive effect of VYE on (**b**) NO secretion and (**c**–**e**) TNF-α, IL-6, and IL-1β cytokine production upon LPS stimulation. RAW 264.7 cells were pretreated with VYE for 1 h prior to incubation with LPS for 24 h. Cytotoxicity was determined by CCK assay. NO content in the conditioned medium was determined using Griess reagent. TNF-α, IL-6, and IL-1β levels in the medium, as measured by ELISA. As a control, cells were incubated with vehicle alone. Data represent the means ± SE of three independent experiments. ** *p* < 0.001 compared to the LPS-stimulated value

To investigate the cytotoxicity of VYE in RAW 264.7 cells, we conducted viability assays using CCK. As shown in Fig. 1a, 10 and 150 μg/mL of VYE had no effect on RAW 264.7 cell viability. Thus, to exclude the potential influence of cytotoxicity, the concentration of VYE was limited to 150 μg/mL in subsequent experiments. Half maximal inhibitory concentration (IC50) value of VYE on the viability of RAW 264.7 cells was 278.8 μg/mL and IC50 of dexamethasone was 390.3 μM (data not shown). Dexamethasone, which is widely used for the treatment of inflammation-related disease, was used as a positive control in this study. Because NO production is related with various inflammatory diseases, we measured the inhibitory activity of VYE on NO production in LPS-stimulated macrophages. The concentration of nitrite in supernatants was determined using Griess reagent. As shown in Fig. 1b, the release of NO was significantly inhibited by VYE pretreatment in a concentration-dependent manner. Especially, 150 μg/mL VYE inhibited NO production by more than 90 %.

### Inhibitory activities of VYE on secretion of inflammatory cytokines and their mRNA expression

**Fig. 2:**
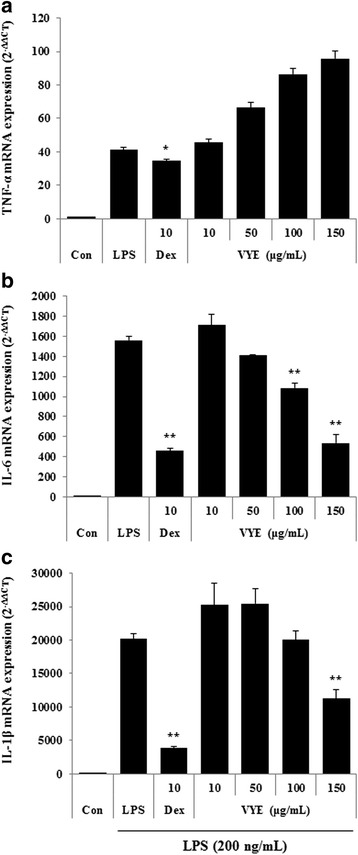
Effect of VYE on LPS-induced (**a**) TNF-α, (**b**) IL-6, and (**c**) IL-1β mRNA expression in macrophages. Cells were pretreated with VYE for 1 h and stimulated with LPS for a further 6 h. mRNA levels were analyzed by real-time RT-PCR. Amplification and analyses of mRNA were performed using the QuantStudio 6 Flex Real-time PCR System (Thermo Scientific, Rockford, IL, USA). Data represent the means ± SE of three independent experiments. * *p* < 0.01 and ** *p* < 0.001 compared to the LPS-stimulated value

To further analyze the potential inhibitory activity of VYE on inflammatory cytokines (TNF-a, IL-6, and IL-1β) production and their mRNAs expression, we used ELISA and real-time RT-PCR. As shown in Fig. 1c, d and e, secretion of IL-6 and IL-1β was significantly repressed by VYE in a dose-dependently. However, VYE had no effect on TNF-α secretion compared with the negative control (LPS treated only) in this assay. Consistent with this result, expression of TNF-α mRNA was not inhibited by treatment with VYE at any concentration (Fig. 2a). However, VYE showed an inhibitory activity on IL-6 and IL-1β mRNA expression at 100 μg/mL or higher (Fig. 2b and c).

### VYE strongly represses iNOS but not COX-2 upon LPS stimulation and induces HO-1 in RAW 264.7 macrophages

**Fig. 3:**
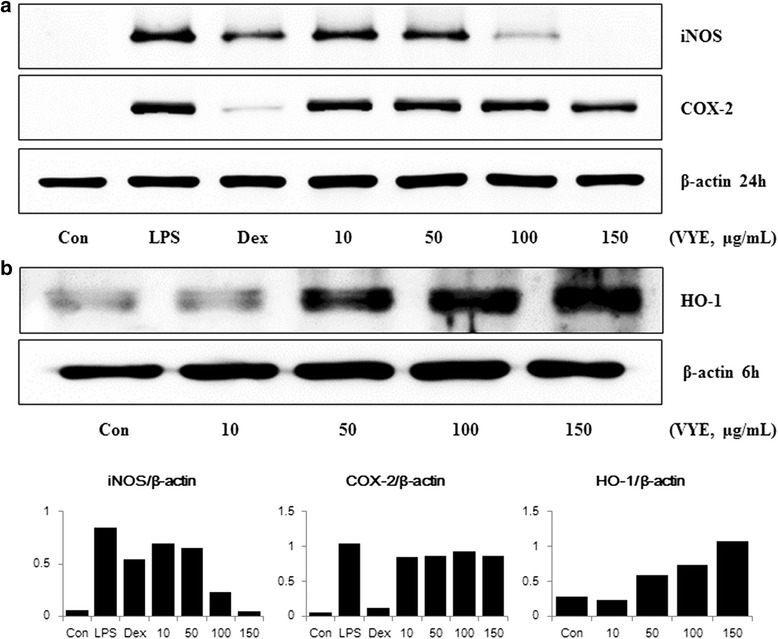
Effect of VYE on (**a**) iNOS, COX-2 protein levels, and (**b**) HO-1 protein induction in RAW 264.7 cells. Cells were treated with (**a**) LPS alone or LPS plus VYE for 24 h or with (**b**) VYE alone for 6 h. Protein levels were determined by Western blotting, as described in the Materials and Methods, and quantitated using a Davinch-chemi™ CAS-400SM chemiluminescence imaging system (Core Bio, Seoul, Korea). β-actin served as a control. The experiment was repeated three times independently, and similar results were obtained

**Fig. 4:**
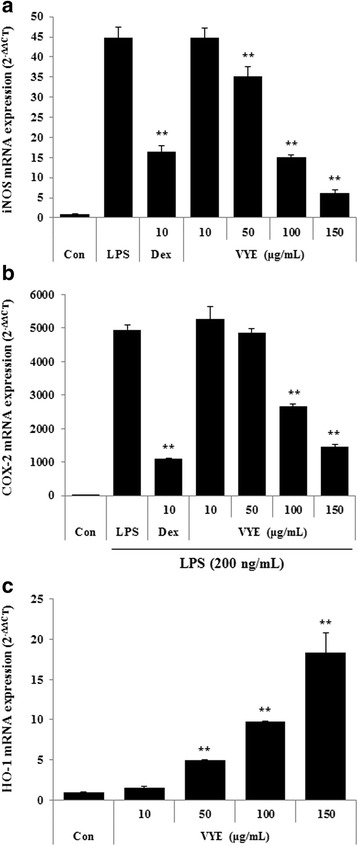
Effects of VYE on mRNA expression of (**a**) iNOS, (**b**) COX-2, and (**c**) HO-1 in macrophages. Cells were treated with (**a**, **b**) LPS alone or with LPS and VYE for 24 h and (**c**) VYE alone for 3 h. Data represent the means ± SE of determinations from three independent experiments. ** *p* < 0.001 compared to the LPS-stimulated value

To examine the mechanism by which VYE reduces NO and cytokine production by LPS stimulation, we evaluated the effect of VYE on iNOS, COX-2, and HO-1 expression in RAW 264.7 macrophages. The inhibitory effects of VYE on the protein and mRNA levels of iNOS, COX-2, and HO-1 were evaluated by Western blot analysis and real-time RT-PCR. As shown in Fig. 3a, VYE strongly reduced protein levels of iNOS. However, COX-2 protein expression was slightly repressed at concentrations of 10–150 μg/mL. In addition, as shown in Fig. 3b, the HO-1 protein level was markedly augmented by VYE treatment. Consistent with this result, VYE treatment significantly repressed the expression of iNOS mRNA and induced that of HO-1 (Fig. 4a and c). By contrast, COX-2 mRNA expression was repressed by VYE treatment, the opposite of the protein levels (Fig. 4b).

### Suppressive effects of VYE on activation of NF-kB signaling pathway in macrophages

**Fig. 5:**
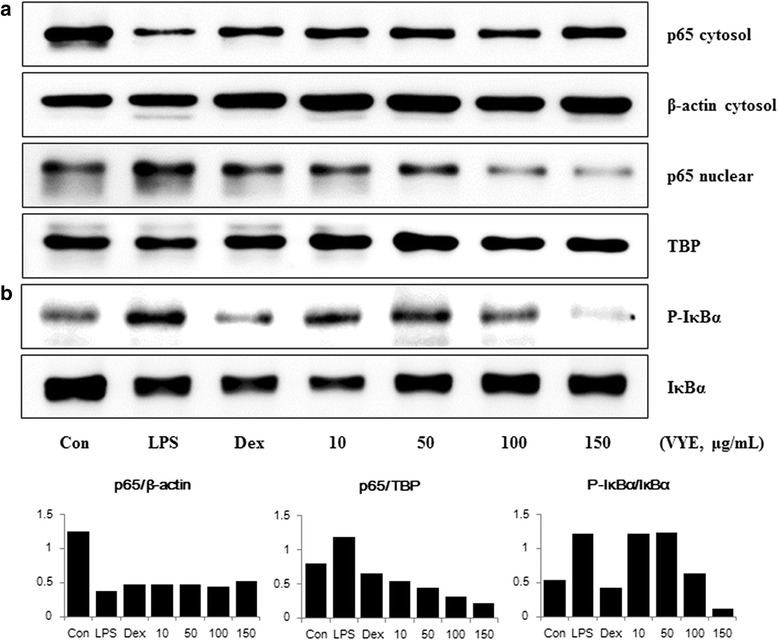
Effects of VYE on LPS-induced (**a**) translocation of NF-kB p65 into the nucleus and (**b**) phosphorylation of IkBα in RAW 264.7 cells. Cells were pretreated with VYE for 1 h and stimulated with LPS for a further (**a**) 1 h or (**b**) 30 min. β-actin and TATA box-binding protein (TBP) were used as control proteins of the cytosolic and nuclear fractions, respectively. The experiment was repeated three times independently, and similar results were obtained

The NF-kB is closely related to inflammatory mediator production such as cytokines and iNOS. Therefore, we assessed the effect of VYE on the activation of NF-kB by analyzing nuclear translocation of p65 and IkBα phosphorylation. As shown in Fig. 5a, VYE significantly inhibited nuclear translocation of NF-kB p65 by LPS stimulation at all concentrations (10–150 μg/mL). We also evaluated the effects of VYE on the phosphorylation and degradation of IkBα upon LPS stimulation. As shown in Fig. 5b, the activation of IkBα through phosphorylation and degradation was reduced in a dose-dependently by VYE treatment. These results suggested that VYE inhibits translocation of NF-kB and phosphorylation, degradation of IkBα.

### VYE inhibits phosphorylation of MAPKs by LPS stimulation in RAW 264.7 cells

**Fig. 6:**
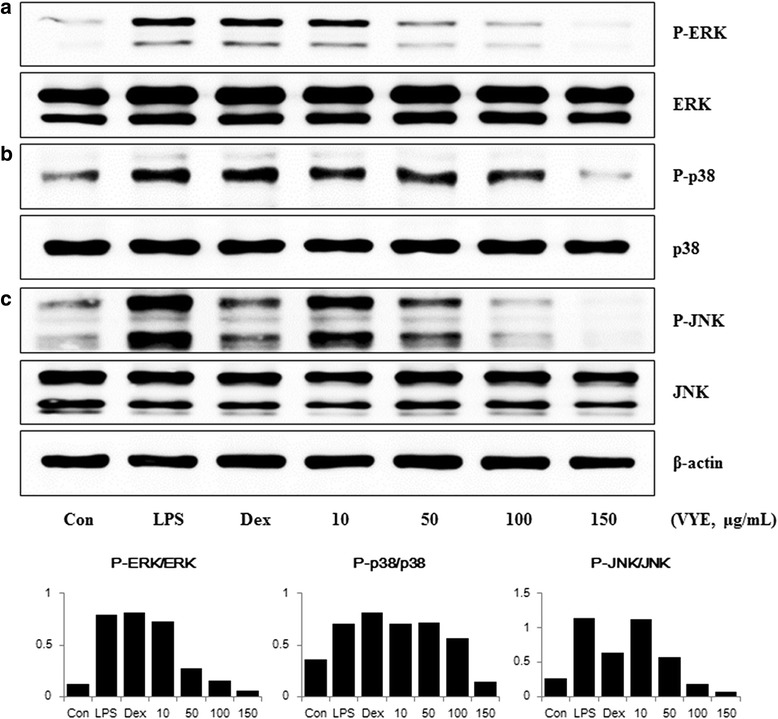
Effects of VYE on the phosphorylation of (**a**) ERK, (**b**) p38, and (**c**) JNK MAPKs in LPS-stimulated macrophages. Cells were treated with VYE for 1 h and challenged with LPS for 30 min. Proteins were examined by Western blot analysis. ERK, p38 and JNK were used as controls for their phosphorylated forms. The experiment was repeated three times independently, and similar results were obtained

The MAPK family members include ERK, p38, and JNK. MAPKs are activated by stimuli such as LPS, and play an essential role in the regulation of inflammatory mediator production. We examined whether activation of MAPKs is repressed by VYE treatment. As shown in Fig. 6a and c, phosphorylation of ERK and JNK MAPK by LPS treatment was markedly decreased in a dose-dependent manner. In addition, VYE only slightly affected p38 activity at concentrations of 10–100 μg/mL, but strongly inhibited phosphorylation of p38 at the highest dose tested (150 μg/mL) (Fig. 6b).

### VYE inhibits cytokine production by LPS stimulation in mouse peritoneal macrophages

**Fig. 7:**
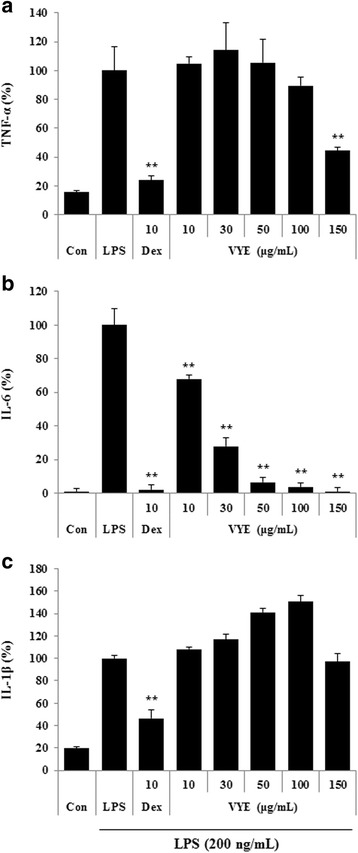
Effect of VYE on LPS-induced (**a**) TNF-α, (**b**) IL-6, and (**c**) IL-1β cytokine production in mouse peritoneal macrophages. Mouse peritoneal macrophage cells were treated with VYE for 1 h prior to incubation with LPS for 24 h. Cytokine levels in the culture supernatant were analyzed by ELISA. As a control, the cells were incubated with vehicle alone. Data represent the means ± SE of three independent experiments. ** *p* < 0.001 compared to the LPS-stimulated value

First, we examined the anti-inflammatory activity of VYE on RAW 264.7 cell line. Next, we confirmed the inhibitory activity of VYE in mouse primary macrophages. We measured the levels of inflammatory cytokines—TNF-α, IL-6, and IL-1β. As shown in Fig. 7b, VYE significantly inhibited secretion of IL-6 in a dose-dependently. However, VYE at concentrations of 10–100 μg/mL only slightly inhibited or did not inhibit TNF-α and IL-1β secretion. In addition, 150 μg/mL VYE suppressed TNF-α and IL-1β production by peritoneal macrophages (Fig. 7a and c). These results indicated that 150 μg/mL VYE effectively inhibited the inflammatory response in primary macrophage cells without toxicity (cell viability data not shown).

### Screening and analyzing bioactive components

**Fig. 8:**
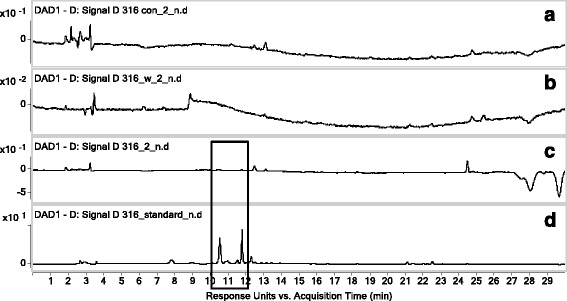
UV chromatogram (330 nm). **a** Desorption eluate of RAW 264.7 cells incubated without VYE, **b** fourth wash eluate of cell extract, **c** desorption eluate of cells incubated with VYE, and **d** extract of VYE

**Fig. 9:**
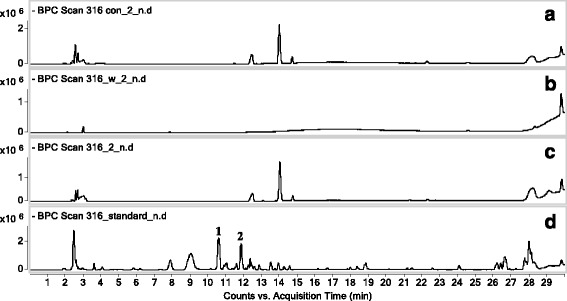
Mass chromatogram (negative mode). **a** Desorption eluate of RAW 264.7 cells incubated without VYE, **b** fourth wash eluate of cell extract, **c** desorption eluate of cells incubated with VYE, and **d** extract of VYE

**Fig. 10:**
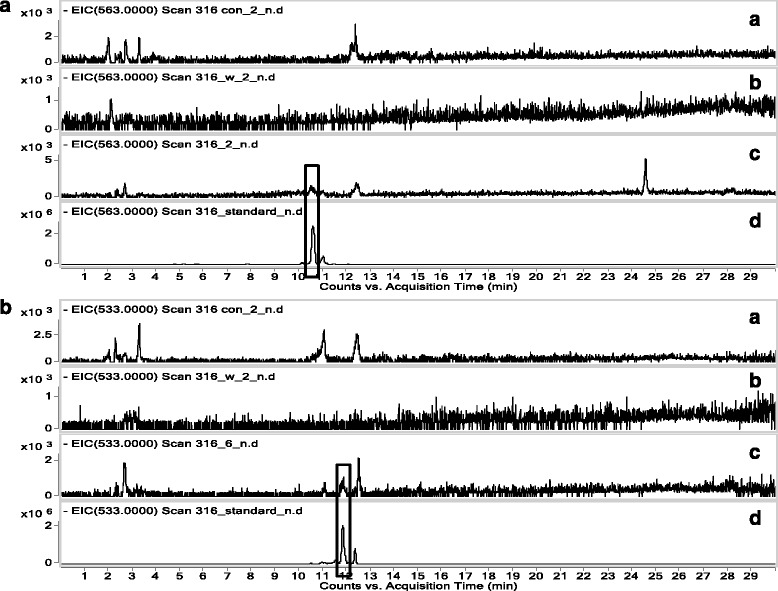
(A) Extracted ion chromatogram (EIC) of VYE extract for 563 and (B) extracted ion chromatogram (EIC) of VYE extract for 533. (A) Desorption eluate of RAW 264.7 cells incubated without VYE, (B) fourth wash eluate of cell extract, (C) desorption eluate of cells incubated with VYE, and (D) extract of VYE

VYE, which has anti-inflammatory effects, was used for screening bioactive candidates that interact with RAW 264.7 cells. The compounds were screened and analyzed using HPLC-DAD-QTOF MS. The UV chromatograms (monitored at 330 nm) of extracts from VYE, blank control (desorption eluate of RAW 264.7 cells incubated without VYE), fourth wash eluate of cell extract, and desorption eluate of cells incubated with VYE are shown in Fig. 8. The chromatogram of VYE extract contained two major peaks at 271 and 338 nm (Fig. 8d). The chromatogram of desorption eluate of cell extract also showed these peaks. However, the chromatogram of the blank control and fourth wash eluate of cell extract did not. The mass chromatograms (monitored negative mode) of extracts from VYE, two major peaks were observed at 10.5 min (*m/z* 563) and 11.8 min (*m/z* 533) (Fig. 9d). Each molecular ion peak at *m/z* 563 and 533 was identified using the extracted ion chromatogram (EIC) mode (Fig. 10a and b). In Fig. 10a and b, the peak was detected only in the desorption eluate of cell extract. In other words, the interaction between components and cells was shown to be highly selective and strong.

### Isolation and identification of the bioactive candidates

**Fig. 11:**
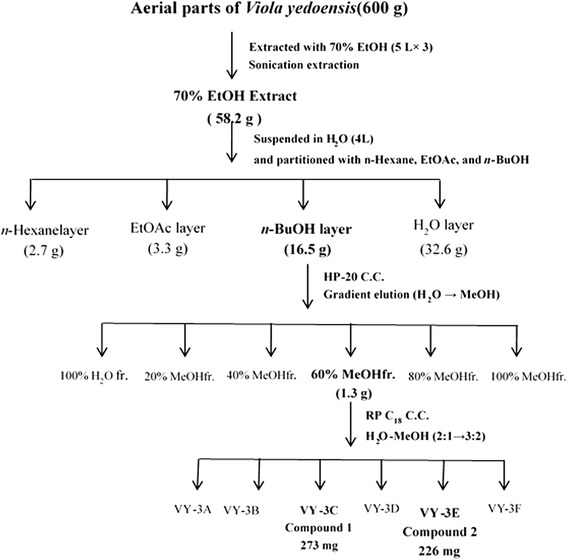
Isolation from *V. yedoensis*

**Fig. 12:**
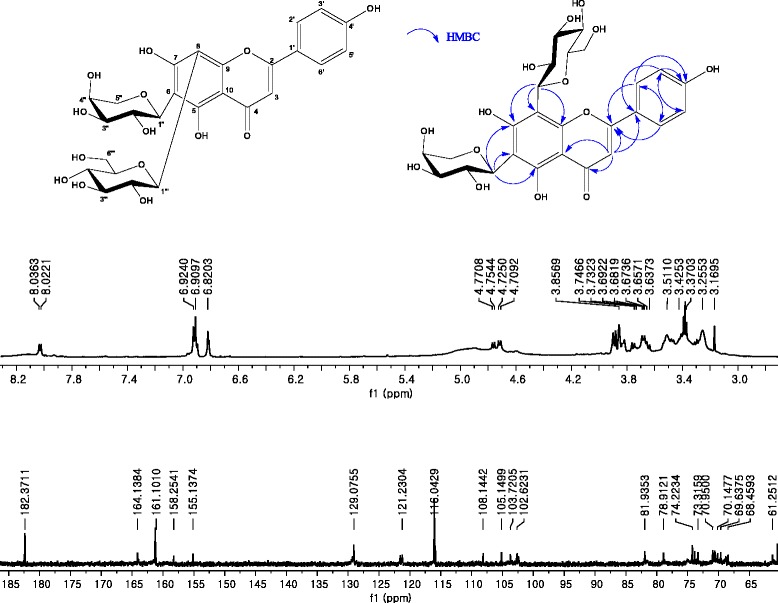
^1^H- and ^13^C-NMR spectra of compound 1

**Fig. 13:**
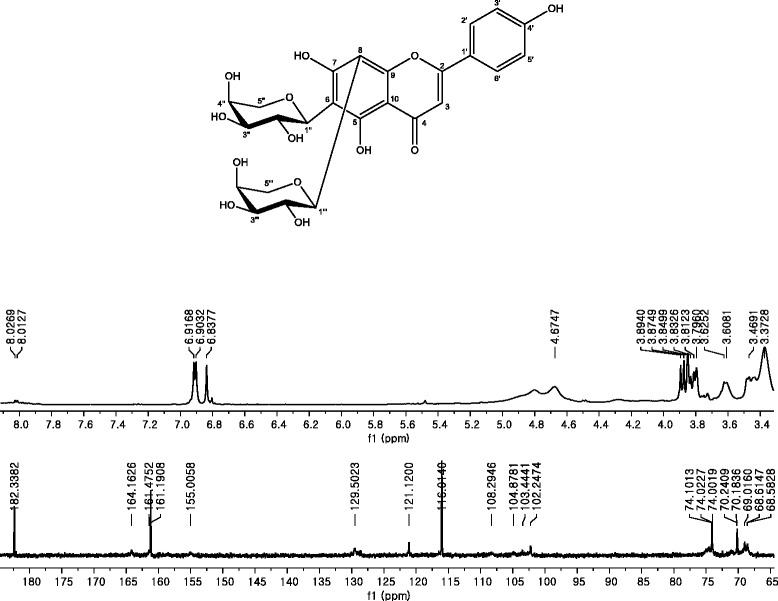
^1^H- and ^13^C-NMR spectra of compound 2

The two compounds of the n-BuOH fraction of VYE were isolated from various open chromatography and TLC profiles (Fig. 11). The structures of two compounds were identified as isoschaftoside (1) [26, 27] and apigenin 6, 8-di-*C*-α-L-arabinopyranoside (2) [27, 28] by spectroscopic analysis of the HPLC-DAD-MS, ^1^H-NMR, and ^13^C-NMR (Figs. 12 and 13 )

### Compound 1

The ^13^C-NMR spectrum of Fig. 12 showed one carbonyl carbon [δ 182.4], 15 aromatic carbons, and typical carbon signals of a glucose moiety. These carbon signals were estimated to be flavonoid glycoside. The ^1^H-NMR spectrum of Fig. 12 showed ortho coupling between δ 6.92 (2H, d, J = 8.5 Hz) and δ 8.03 (2H, d, J = 8.5 Hz) and two anomeric protons [δ 4.72 and δ 4.76]. The molecular formula was determined to be C_26_H_28_O_14_ based on the ESI-MS (*m/z* 563.1441 [M-H]^−^). Consequently, the chemical structure of compound 1 was elucidated as apigenin 6-*C*-α-L-arabinopyranosyl-8-C-β-D-glucopyranpside, which was named isoschaftoside.

### Compound 2

The ^13^C-NMR spectrum of Fig. 13 contained one carbonyl carbon [δ 182.3], 15 aromatic carbons, and typical carbon signals of the glucose moiety, similar to compound 1. Therefore, the compound was estimated to be flavonoid glycoside. The ^1^H-NMR spectrum of Fig. 13 showed ortho coupling between δ 6.91 (2H, d, J = 8.4 Hz) and δ 8.02 (2H, d, J = 8.4 Hz), and two anomeric protons [δ 4.70]. Its molecular ion peak was at *m/z* 533.1363 [M-H]^−^in the ESI-MS. The molecular formula was deduced as C_25_H_26_O_13_ on the basic ^1^H-, ^13^C-NMR, and ESI-MS data. Thus, the chemical structure of compound 2 was determined to be apigenin 6, 8-di-*C*-α-L-arabinopyranoside.

## Discussion

VY is an important medicinal herb in traditional medicine and has been used to treat associated with inflammation-related diseases in Korea [1]. A previous study revealed that VY exerts anti-HIV effects [2], and another revealed anti-coagulant activity [3]. In addition, recent studies have shown the protective effect of VY on LPS-induced acute lung injury in mice [4].

In our present study, we evaluated the anti-inflammatory effect of VYE in LPS-induced RAW 264.7 cells and mouse primary macrophages. First, we investigated that VYE treatment did not result in cytotoxicity to macrophages, did not affect cell viability at concentrations below 150 μg/mL. Overproduction of NO and iNOS is closely related to lots of inflammatory diseases [29, 30]; thus, we examined the suppress effects of VYE on NO and iNOS production induced by LPS. We found that VYE strongly inhibited NO secretion and repressed expression of iNOS protein and mRNA levels, which is the synthesizing enzyme of NO, in a concentration-dependent manner. These results indicate that the suppressive effect of VYE on iNOS expression conduce to the inhibition of NO secretion.

The expression of NO and iNOS was due to a direct influence on the induction of HO-1 [31], we further investigated the effect of VYE treatment on HO-1 expression. Thus, we determined the HO-1 protein and mRNA expression by VYE treatment absence of LPS. In our experiments, VYE exerts strongly induced HO-1 expression dose-dependently (10–150 μg/mL) in macrophage RAW 264.7, also the production of HO-1 influenced the inhibitory activity of VYE on NO secretion and iNOS expression. This result about HO-1 expression suggests that the anti-inflammatory effects of VYE is influenced not only by a blockade of NF-kB and MAPK pathway activation but also by the induction of HO-1.

VYE inhibited the secretion of inflammatory cytokines and expression of their mRNA genes, such as IL-6 and IL-1β. The transcription factor NF-kB is an important regulator of various genes involved in inflammatory responses [32]. After activation of NF-kB by LPS, IkB bound to NF-kB in cytoplasm becomes phosphorylated, leading to p65 translocation into the nucleus and phosphorylation of IkBα. Thus, we examined p65 levels in cytoplasmic and nuclear extracts by Western blotting. As a result, VYE reduced translocation and degradation of NF-kB p65, phosphorylation of IkBα upon LPS stimulation in a dose-dependent manner. These data are consistent with previous studies that indicated that NF-kB response elements are reveal in the promoters of the genes encoding iNOS and inflammatory cytokine [16, 33–35]. Since many anti-inflammatory drugs inhibit NF-kB activation, VYE extract could represent a novel anti-inflammatory agents.

MAPKs activated by LPS are associated with production of inflammatory mediators such as iNOS expression in RAW 264.7 cells [36], we also investigated the inhibitory effect of VYE on MAPK phosphorylation. Phosphorylation of ERK and JNK MAPK were significantly inhibited, but had only a minor effect on the phosphorylation of p38 MAPK by VYE treatment. These results mean that the anti-inflammatory activity of VYE on MAPKs phosphorylation is directly involved in the inhibition of NF-kB activation and repression of inflammatory mediator production in macrophages. In this study, we examined whether VYE inhibits various inflammatory mechanisms, such as NF-kB, MAPK, and HO-1. We found that VYE shows strong inhibitory effects on various signaling pathways.

We further investigated the inhibitory activity of VYE on cytokine production in mouse peritoneal macrophages upon LPS stimulation to confirm its anti-inflammatory effect. Consistent with the results from cell lines, VYE significantly inhibited the production of IL-6 cytokines in primary cells without exerting cytotoxicity. However, it did not inhibit TNF-α and IL-1β production, except at the highest concentration tested (150 μg/mL). These results suggest that VYE inhibits the inflammatory response not only in RAW 264.7 cells but also in primary cells.

In this study, we examined the anti-inflammatory activity and inhibitory mechanism of VYE. Numerous assays showed that VYE inhibited the production of inflammatory mediators including NO and pro-inflammatory cytokines such as TNF-a, IL-6 and IL-1β, as well as activation of the NF-kB and MAPK signaling pathways. Moreover, expression of HO-1 was strongly induced by VYE treatment. These results indicate that VYE has suppressive effects on inflammatory responses. Based on these results, we screened the potential bioactive components of VYE in macrophages by LC-MS and determined their chemical structure using HPLC-DAD-MS, ^1^H-NMR, and ^13^C-NMR. We identified two major bioactive components that could interact with macrophages and investigated the structures of these compounds. The chemical structure of compound 1 was apigenin 6-*C*-α-arabinopyranosyl-8-*C*-β-D-glucopyranpside, called isoschaftoside. The chemical structure of compound 2 was apigenin 6, 8-di-C-α-L-arabino-pyranoside.

In summary, we demonstrated the anti-inflammatory activity and inhibitory mechanism of VYE. We also identified two bioactive components that interact with macrophages and determined their structures. The role in inflammation of the two pure compounds warrants further studies.

## Conclusions

In conclusion, the results of the present study demonstrated that VYE has strong inhibitory activity on production of inflammatory mediators including NO, inflammatory cytokines, and iNOS in LPS-stimulated RAW 264.7 cells. These effects are mediated by inhibiting NF-kB activation through IkBα stabilization and blocking MAPK phosphorylation. In addition, induction of HO-1 expression by VYE involved in the inhibition of inflammatory mediators, and VYE inhibited the production of inflammatory cytokines in mouse primary macrophages. Furthermore, we screened two bioactive components and determined their chemical structure. These results indicate VYE to have potential as an anti-inflammatory agent.

## Abbreviations

ATCC, American Type Culture Collection; BSA, bovine serum albumin; CCK, cell counting kit; COX, cyclooxygenase; EIC, extracted ion chromatogram; ELISA, enzyme-linked immunesorbent assay; ERK, extracellular signal-regulated kinase; ESI, electrospray ionization; FBS, fetal bovine serum; HO, heme oxygenase; HRP, horseradish peroxidase; IL, interleukin; iNOS, inducible nitric oxide synthase; IkBα, inhibitors of NF-kB alpha; JNK, Jun NH_2_-terminal kinase; LC-MS, high-performance liquid chromatography tandem mass spectrometry; LPS, lipopolysaccharide; MAPK, mitogen-activated protein kinase; NC, nitrocellulose; NF, nuclear factor; NO, nitric oxide; PBS, phosphate-buffered saline; RBC, red blood cell; RIPA, radio immunoprecipitation assay; RPMI, Roswell Park Memorial Institute; RT, room temperature; SDS-PAGE, sodium dodecyl sulfate-polyacrylamide gel electrophoresis; TNF, tumor necrosis factor; VY, *Viola yedoensis*; VYE, VY ethanol extract.
